# Diagnosis of helminths depends on worm fecundity and the distribution of parasites within hosts

**DOI:** 10.1098/rspb.2022.2204

**Published:** 2023-01-25

**Authors:** Thomas Crellen, Melissa Haswell, Paiboon Sithithaworn, Somphou Sayasone, Peter Odermatt, Poppy H. L. Lamberton, Simon E. F. Spencer, T. Déirdre Hollingsworth

**Affiliations:** ^1^ School of Biodiversity One Health and Veterinary Medicine, Graham Kerr Building, University of Glasgow, 82 Hillhead Street, Glasgow G12 8QQ, UK; ^2^ Wellcome Centre for Integrative Parasitology, Sir Graeme Davies Building, University of Glasgow, 120 University Place, Glasgow G12 8TA, UK; ^3^ Big Data Institute, Li Ka Shing Centre for Health Information and Discovery, University of Oxford, Oxford OX3 7LF, UK; ^4^ Office of the Deputy Vice Chancellor, Indigenous Strategy and Services and School of Geosciences, John Woolley Building, University of Sydney, Sydney, New South Wales 2050, Australia; ^5^ School of Public Health and Social Work, Kelvin Grove Campus, Queensland University of Technology, Brisbane City, Queensland 4000, Australia; ^6^ Department of Parasitology, Khon Kaen University, 123 Thanon Mittraphap, Khon Kaen 40002, Thailand; ^7^ Lao Tropical and Public Health Institute, Samsenthai Road, Sisattanak district, Vientiane, Lao PDR; ^8^ Department of Public Health and Epidemiology, Swiss Tropical and Public Health Institute, Kreuzstrasse 2, Allschwil 4123, Switzerland; ^9^ University of Basel, Petersplatz 1, Basel 4001, Switzerland; ^10^ Department of Statistics, University of Warwick, Coventry CV4 7AL, UK

**Keywords:** neglected tropical diseases, foodborne trematodiases, epidemiology, macroparasite, Bayesian inference

## Abstract

Helminth transmission and morbidity are dependent on the number of mature parasites within a host; however, observing adult worms is impossible for many natural infections. An outstanding challenge is therefore relating routine diagnostics, such as faecal egg counts, to the underlying worm burden. This relationship is complicated by density-dependent fecundity (egg output per worm reduces due to crowding at high burdens) and the skewed distribution of parasites (majority of helminths aggregated in a small fraction of hosts). We address these questions for the carcinogenic liver fluke *Opisthorchis viverrini*, which infects approximately 10 million people across Southeast Asia, by analysing five epidemiological surveys (*n* = 641) where adult flukes were recovered. Using a mechanistic model, we show that parasite fecundity varies between populations, with surveys from Thailand and Laos demonstrating distinct patterns of egg output and density-dependence. As the probability of observing faecal eggs increases with the number of mature parasites within a host, we quantify diagnostic sensitivity as a function of the worm burden and find that greater than 50% of cases are misdiagnosed as false negative in communities close to elimination. Finally, we demonstrate that the relationship between observed prevalence from routine diagnostics and true prevalence is nonlinear and strongly influenced by parasite aggregation.

## 1. Introduction

Parasitic helminths continue to cause diseases of major medical and veterinary significance. The frequency distribution of mature parasites is the key quantity of interest in the epidemiology of helminths as higher worm burdens are linked with increased host infectiousness, more severe morbidity, and are a marker of past exposure [1–3]. The transmission dynamics of helminths, also classified as macroparasites, can be described through changes to the mean worm burden in the population over time. This is in contrast to many protozoal, bacterial and viral infections (collectively known as microparasites), where the trajectory of epidemics can be characterized by the number of hosts in certain disease states; for instance susceptible, infected or recovered [2,4]. In populations of humans [2] and wildlife [5], helminths are typically aggregated in a small fraction of hosts. This makes control using mass deworming more challenging compared to randomly distributed parasites [6], however if heavily infected hosts can be identified it presents an opportunity to drive down transmission rates. Worm burden is also a critical driver of chronic morbidity. While the mechanisms for helminth-induced pathology are species-specific, in many cases, chronic damage is attributable to the intensity and duration of past infection with adult worms [7].

Despite the importance of characterizing the distribution of parasites within a population, it is challenging to directly observe mature worms from natural hosts for most species of helminths, with the roundworm *Ascaris lumbricoides* as a notable exception [8–10]. Diagnosis typically relies on indirect coprological or serological measures of the worm burden, such as faecal egg counts, worm antigen concentrations or molecular detection of parasite DNA in blood or stool. Historical autopsies provide critical insights into the relationship between worm burdens and indirect diagnostics for several helminth species [11–13]; however, these studies may not be reflective of current parasite fecundity in endemic settings due to host population heterogeneity. For instance, the fecundity of *A. lumbricoides* differs markedly between hosts in Bangladesh and Nigeria [14]. The relationship may also alter over time as the nutritional status of populations changes and control programmes reduce the intensity of parasite infections through repeated mass treatment [15,16]. In the absence of data on worm burdens, statistical frameworks have been proposed to infer the number of adult worms at the population level from indirect diagnostics, usually counts of parasite eggs in stools [17–19]. However, accurate inference of the worm burden is dependent on substantial prior knowledge of parasite ecology including (i) the relationship between worm burden and egg output in the population of interest; (ii) a probability distribution which captures the variability in egg output from a given number of worms, and (iii) the sensitivity and specificity of diagnostics.

The liver fluke *Opisthorchis viverrini* infects around 10 million people across Southeast Asia, predominantly in Laos, Cambodia and the North and Northeast regions of Thailand [20]. The parasite has a complex life cycle which includes both *Bithynia* snails and freshwater fish as intermediate hosts. Humans become infected by consuming raw or undercooked fish encysted with metacercariae. After ingestion, adult worms migrate to the bile duct where they cause chronic damage to host tissue through a combination of mechanical damage, inflammation and the secretion of proteins; most notably the cell growth factor granulin [21,22]. The pathology progresses to cholangiocarcinoma (bile duct cancer) in around 1–5% of infected people, with higher worm burdens increasing the risk of developing cancer [23,24]. Humans do not develop immunity to *O. viverrini* and repeated reinfection is common [25]. Nevertheless, regular anthelmintic treatment to kill adult worms is likely to slow the progression of liver pathology and reduce the risk of developing cholangiocarcinoma [26]. The resulting sequela is considered a neglected tropical disease (NTD), under the grouping of foodborne trematodiases, and is targeted for enhanced control globally by 2030 [27]. Endemic countries have initiated control programmes, with Thailand the first country to start an opisthorchiasis control programme in 1950, aiming to halt parasite transmission and reduce cases of the resulting cholangiocarcinoma [28]. Central to achieving these objectives is a quantitative understanding of parasite transmission to inform control strategies [29,30].

**Figure 1.**
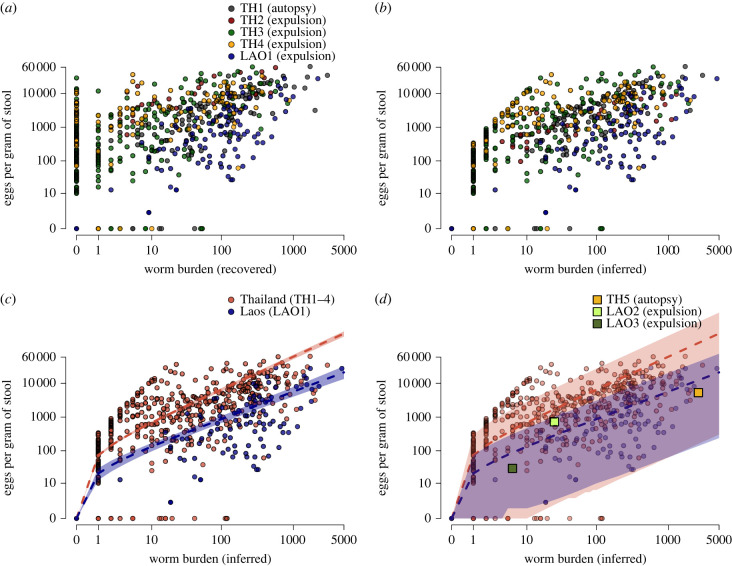
Observed data on *Opisthorchis viverrini*, worm burden inference and parasite fecundity. (*a*) Relationship between observed *O. viverrini* adult worms (*w*) and eggs per gram of stool (*y*) in five surveys (table 1) shown on the log scale. (*b*) Relationship between inferred worm burdens (*x*) and faecal egg counts. Eggs per gram for TH1 and TH2 have been adjusted by the model. (*c*) As (*b*) with points coloured by country of survey. The dashed lines are a power law function with parameter values taken from mean posterior estimates. The shaded area indicates the 95% credible interval of the mean. (*d*) As (*c*) with the shaded area representing the 90% prediction interval based on a zero-truncated negative binomial error distribution. Validation data are shown as square points (table 1).

The overdispersed pattern of helminth parasites in a population is best captured with a negative binomial distribution, also known as a gamma–Poisson mixture, parameterized by the mean worm burden *M* and a dispersion factor *k* that scales inversely with the variance [2]. While previous studies have reported an aggregated distribution of *O. viverrini* worms within human hosts [31–33], the negative binomial distribution has never been fitted to data on *O. viverrini* and therefore there are no likelihood-based estimates of the aggregation parameter *k* [30]. The relationship between *O. viverrini* adult worm burdens and faecal egg counts has previously been reported from either (i) autopsies in which the liver is dissected, or (ii) surveys where worms are expelled by administration of a saline purgative following anthelmintic treatment with praziquantel (table 1 and figure 1*a*). Typically, a linear regression between log-transformed worm burden (*x*) and total egg counts per host (*y*) is used; log_10_(*y* + 1) = *β* · log_10_(*x* + 1), or $y={\left(x+1\right)}^{\beta }-1$ when untransformed. Previous values of *β* are 1.67 [34] and 2.0 [31] which indicate inverse density-dependence. As this is biologically implausible and contrary to the reported findings in these studies, the log-log linear model is likely inappropriate. Alternative parametric functions have been proposed to model the relationship between total worm burden and egg counts, including power law [40] and algebraic decay functions [41].

**Table 1.** Cross-sectional surveys investigating the relationship between adult *Opisthorchis viverrini* worm burden and faecal egg counts in humans included in the analysis. Surveys TH1–4 and LAO1 had individual-level data used for model fitting. Surveys TH5 and LAO2–3 had aggregated data used for model validation. The table shows the method of adult worm collection, either from liver examination during autopsy or expulsion with a saline purgative after anthelmintic treatment with praziquantel; the study location and date; the number of human participants (sample size); the arithmetic mean number of *O. viverrini* adult worms recovered; and the diagnostic method used for parasite egg identification and counting. TH relates to surveys in Thailand and LAO to Laos.

survey and method	location and year(s)	sample size	worms recovered			faecal egg diagnostic
			mean	range	s.d.^a^	
TH1 autopsy [34]	Khon Kaen 1982–1999	139	160	0–2954	390	FECT^b^
TH2 expulsion [35]	Khon Kaen 1987	33	85	0–565	154	Stoll’s dilution
TH3 expulsion [31]	Kalasin 1989	231	39	0–832	105	FECT^b^
TH4 expulsion [36]	Khon Kaen 1991	141	49	0–874	102	FECT^b^
LAO1 expulsion [37]	Savannakhet 2005	97	183	0–2178	286	FECT^b^
TH5 autopsy^c^ [38]	Khon Kaen 1966	9	2588	20–11856	NA^d^	Stoll’s dilution
LAO2 expulsion^c^ [39]	Savannakhet 2008	125	11	0–111	29	Kato–Katz
LAO3 expulsion^c^ [39]	Savannakhet 2011	82	3	0–66	9	Kato–Katz

^a^Standard deviation.

^b^Formalin–ether concentration technique.

^c^Survey used for validation only.

^d^Data not available.

The sensitivity of parasitological diagnostics is known to covary with the number of mature worms within hosts (or pairs of mated adult worms in the case of dioecious helminths) as the probability of observing at least one egg in stool or urine increases with higher worm burdens [42]. Diagnostic sensitivity is rarely considered as a function of worm burden, however, this provides considerable insights into the relationship between the true and observed prevalence and how the reliability of diagnostics evolves as control programmes reduce infection intensity [43,44].

**Figure 2.**
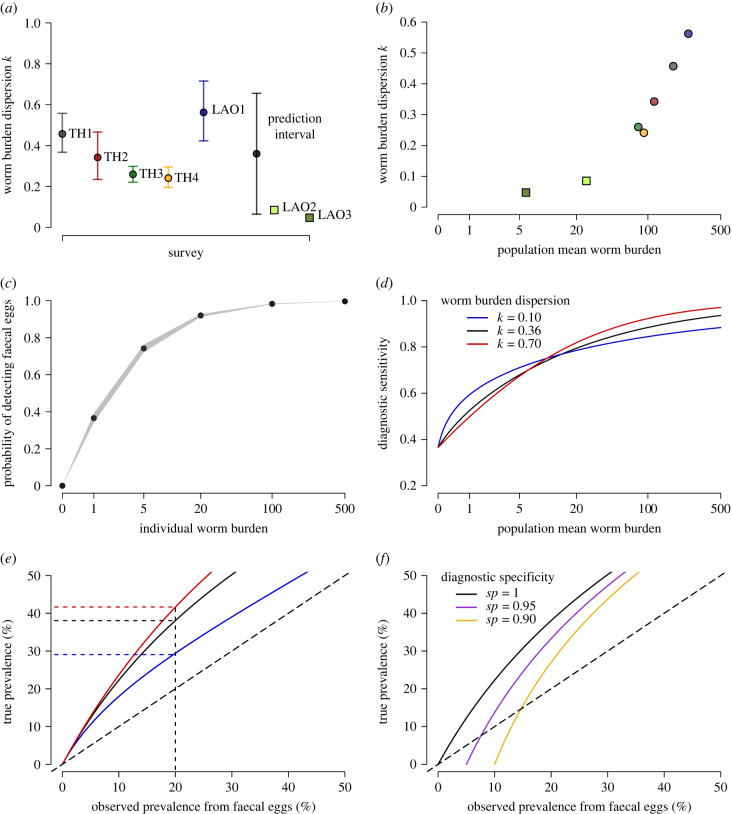
Distribution of adult *O. viverrini* worms and diagnostic sensitivity. (*a*) Negative binomial dispersion parameter *k* by survey (table 1) where points show the posterior mean and lines the 95% credible interval, 90% prediction interval, and validation datasets shown as squares. (*b*) Relationship between estimates of *k* and the estimated mean worm burden (*M*) by survey. (*c*) The probability of detecting ≥ 1 faecal egg by parasitological diagnostic (formalin–ether concentration technique; FECT) as a function of an individual’s worm burden (*x*), the shaded area gives the 95% credible interval. (*d*) Sensitivity of parasitological diagnostics (FECT) at the population level for different frequency distributions of adult worms, given by the aggregation parameter *k*. (*e*) Relationship between the observed prevalence by parasitological diagnostic (FECT) and the true prevalence for different values of the aggregation parameter *k* (see legend in (*d*)). Long dashed line gives equality between axes. Short dashed lines indicate the true prevalence when the observed prevalence is 20%. (*f*) As (*e*) with a varying specificity of parasitological diagnostic, where worm aggregation *k* = 0.36 for all curves.

Here, we infer the *O. viverrini* worm burden at an individual and community level (figure 1*b*) using a mechanistic statistical model. We quantify the relationship between worm burden and egg output in separate populations (figure 1*c*); provide the first fitting of the negative binomial distribution to *O. viverrini* worms at the population level using partial pooling across surveys to estimate the aggregation parameter *k* (figure 2*a*); estimate the sensitivity of faecal egg diagnostics as a function of the worm burden (figure 2*c*,*d*); and show the relationship between the true and observed prevalence (figure 2*e*,*f*). Our study provides a foundation for accurately characterizing the infection intensity of helminths in endemic communities and estimating epidemiological parameters to facilitate evidence-based control.

## 2. Material and methods

### (a) Epidemiological framework

We developed and applied a mechanistic statistical model to individual-level data on adult worm burdens and egg counts which incorporates key aspects of parasite ecology. Our observed data are the counts of recovered *O. viverrini* adult worms from each individual *i* in survey *j*, denoted *w*_*ij*_, and the eggs per gram of stool, denoted *y*_*ij*_. We start from the assumption that the true number of *O. viverrini* adult worms per individual, *x*_*ij*_, follow the negative binomial distribution within community *j*, which is parameterized by a mean worm burden *M*_*j*_ and dispersion factor *k*_*j*_ [2,5]

2.1 $$\mathrm{Pr}(X_{ij}=x|k_{j},M_{j})=\left(\begin{array}{c} x+k_{j}-1\\ x \end{array}\right){\left(\frac{k_{j}}{M_{j}+k_{j}}\right)}^{k_{j}}{\left(\frac{M_{j}}{M_{j}+k_{j}}\right)}^{x}.$$

The value of *k*_*j*_ from each study is themselves normally distributed with an overall mean of *μ*_*k*_ and a standard deviation of *σ*_*k*_, meaning that estimates of *k*_*j*_ are informed by partial pooling. In worm expulsion studies, participants are treated with the anthelmintic praziquantel followed by a saline purgative, either magnesium sulfate solution or sodium sulfate solution, several hours later. This is known to result in imperfect recovery as worms can be degraded and unrecoverable following anthelmintic treatment; remain blocked in the biliary tracked; or stool sample collections may be missed [45,46]. Therefore, we allow the true worm burden to be greater or equal to the observed number of worms for individuals in expulsion studies (*x*_*ij*_ ≥ *w*_*ij*_) and the probability of observing *w*_*ij*_ worms given a true count of *x*_*ij*_ is a binomial sampling process with a probability of worm recovery *r* that is constant across surveys. During autopsy, adult *O. viverrini* were carefully removed from cross sections of liver and worm recovery is likely close to 100% [34]. Therefore, we consider that the true worm count for each individual is equal to the recovered worms in autopsy studies (*x*_*ij*_ = *w*_*ij*_, *r* = 1).

The expected *O. viverrini* eggs per gram of stool for each person, *π*_*ij*_, is given by the generalized function Λ(x,θ), where *θ* is the set of parameters specific to that function. Here, we show results from the power law function as the impact of density-dependent regulation on helminth fecundity is directly quantified by the *γ* parameter; $\pi_{ij}={\left(\lambda_{c(j)}x_{ij}\right)}^{\gamma_{c(j)}}$, where *c*(*j*) denotes the country of survey *j*. As *O. viverrini* is hermaphroditic [47], we assume that a single worm is capable of producing eggs. Observed egg counts for an individual, *y*_*ij*_, are a realization of an error distribution with mean *π*_*ij*_. Our model uses a negative binomial hurdle distribution with dispersion parameter *h*_*c*(*j*)_ and a separate function for false-negative egg counts, which gives the probability of observing zero eggs as a function of the worm burden within an individual

2.2 $$\begin{array}{rl} & \mathrm{Pr}(Y_{ij}=y|X_{ij}=x,\pi_{ij},h_{c(j)},b)\\ & =\left\{ \begin{array}{cc} 1 & y=0,x=0\\ 0 & y\geq 1,x=0\\ 1-\frac{x}{x+b} & y=0,x\geq 1\\ \frac{x}{x+b}\left(\begin{array}{c} y+h-1\\ y \end{array}\right)\frac{{\left(h/(\pi +h)\right)}^{h}{\left(\pi /(\pi +h)\right)}^{y}}{1-{\left(h/(\pi +h)\right)}^{h}} & y\geq 1,x\geq 1. \end{array}\right. \end{array}$$

Bringing these probabilities together, we obtain an expression for the likelihood

2.3 $$\begin{array}{cc} & \mathrm{Pr}(Y_{ij}=y,W_{ij}=w|\pi_{ij},h_{c(j)},b,r,M_{j},k_{j})\\ & \;=\sum_{x=w}^{\infty }\mathrm{Pr}(Y_{ij}=y|X_{ij}=x,\pi_{ij},h_{c(j)},b)\cdot \\ \; & \mathrm{Pr}(W_{ij}=w|x,r)\cdot \mathrm{Pr}(X_{ij}=x|M_{j},k_{j}). \end{array}$$

The diagnostic sensitivity *S* varies by survey *j*, and is dependent on individual sensitivity, or the probability of observing at least one faecal egg as a function of an individual’s worm burden *se*(*x*) = *x*/(*x* + *b*), and the frequency distribution of worms in the community

2.4 S(M,k)=∑x=1∞se(x)(x+k−1x)(k/(M+k))k(M/(M+k)xp(M, k),

where *p*(*M*, *k*) indicates the true prevalence and is given by

2.5 $$p(M,k)=1-{\left(\frac{k}{M+k}\right)}^{k}.$$

The observed prevalence *p*′ for survey *j* is therefore

2.6 $$p^{\prime }=p(M,k)\cdot S(M,k)+\left(1-p(M,k)\right)\cdot (1-sp),$$

where *sp* gives the diagnostic specificity.

### (b) Data sources

We accessed individual-level data from one autopsy (*n* = 139) and four worm expulsion surveys (*n* = 502), which are summarized in table 1. We contacted the study authors to access the original data. Where these were unavailable, we extracted data from publication figures using webplotdigitizer (v. 4.5) and obtained additional information, e.g. the number of (0, 0) points, from text and tables. We confirmed that extracted data showed near identical values to the original reports. Two studies used different methodologies to transform observed egg counts into the reported eggs per gram of stool (EPG). In survey TH1 egg counts were multiplied by an additional factor between 1 and 4 depending on stool consistency [34]. As this was not performed in other surveys, we introduced an integer factor for each individual in TH1, *ξ*_*i*_, which can take on values ∈{1, 2, 3, 4} and the expected egg count is modified by this factor; $\pi_{i}=\Lambda (x,\theta )\xi_{i}$. Another survey (TH2) used an older technique, Stoll’s dilution, to calculate EPG. We, therefore, modified the expected egg counts from TH2 with a real factor; π=Λ(x,θ)ρ, to ensure that our estimates of diagnostic parameters are consistent for FECT. We obtained aggregated data from a further three surveys where *O. viverrini* worms were recovered, which were used for model validation (TH5, LAO2 and LAO3; see table 1). For the expulsion surveys (LAO2 and LAO3), we corrected the mean number of worms recovered by dividing the observed values by the probability of worm recovery (*r*) estimated in the model. Values of *k* for LAO2 and LAO3 (figure 2*a*,*b*) were approximated using the negative binomial relationship between prevalence and corrected mean worm burden (equation (2.5)). Egg counts in TH5 were reported as perworm per day (3160), which was converted to eggs per gram of stool by multiplying by the mean number of worms, then dividing by the expected daily mass of stool (250 g in developing countries [48]) and the correction factor for Stoll’s dilution (*ρ*).

### (c) Model fitting

We fitted statistical models to data using Hamiltonian Monte Carlo implemented in Stan v. 2.30.1 [49,50]. Integer parameters were marginalized out of the likelihood function (equation 2.3) and estimated by computing the normalized likelihood. As the model fitting was performed in a Bayesian framework, parameters were assigned weakly or moderately informative prior distributions based on the available literature. Each model was run with four parallel chains with a burn-in of 2750 iterations per chain and a total of 1000 samples from the posterior distribution. Model fitting diagnostics used to determine successful chain convergence were the Gelman–Rubin diagnostic $\hat{r}\leq 1.01$ and effective sample size greater than 500 [51]. We report the posterior mean and 95% credible interval (CrI) for estimated parameters. Data were processed and figures produced in R v. 4.2.1 [52].

## 3. Results

### (a) Individual worm burdens

Expulsion of liver flukes following anthelmintic treatment is known to result in imperfect recovery [31,45] and we quantified the proportion of adult flukes recovered as less than half (*r* = 0.44 [95% CrI 0.42–0.45]). The true *O. viverrini* worm burden for each individual (*x*_*ij*_) was estimated as greater than the observed count (*w*_*ij*_) for 421/502 people in worm expulsion studies, with a median increase of 10 worms in the inferred burden and a mean increase of 92 worms (figure 1*b*). Across all positive cases for *O. viverrini,* the inferred adult worm burden ranges from 1 to 4677 with a median of 38 and a mean of 192.

### (b) Worm fecundity and variance in faecal egg counts

The expected parasite fecundity, measured as eggs per gram of stool, was quantified using a power law function (see Methods), which directly estimates the number of eggs produced by a single fluke (*λ*), and the extent of density-dependence (*γ*), where values *γ* < 1 indicate a decline in *per capita* egg output at higher worm burdens. From surveys in Thailand, one adult *O. viverrini* was estimated to produce a mean of 72 eggs per gram of stool (95% CrI 66–78), which is substantially higher than the 20 eggs per gram of stool (95% CrI 13–30) estimated from a single fluke in Laos. Our results suggest that density-dependence regulates egg output at higher worm burdens in Laos (*γ* = 0.82 [95% CrI 0.74–0.90]) and to a lesser extent in Thailand, where the posterior mean for *γ* was close to unity (*γ* = 0.98 [95% CrI 0.96–0.99]), see figure 1*c*. The functional relationship between worm burden (*x*) and expected egg output (*π*) is therefore *π* = 72*x*^−0.98^ in Thailand and *π* = 20*x*^−0.82^ in Laos. Expected eggs per gram of stool for survey TH2, which used an alternative diagnostic method (table 1), were corrected by a factor estimated in the model (*ρ* = 6.2). There was substantial variation around the expected values for egg counts, which is consistent with reports from other helminths [53]. We quantified this variance using a zero-truncated negative binomial distribution, also known as a hurdle model (equation (2.2)), characterized by a mean and an aggregation parameter *h*, which scales inversely with variance. Estimated values of *h* for Thailand (*h* = 0.40 [95% CrI 0.32–0.48]) indicate substantially higher variation in egg output compared with Laos (*h* = 0.63 [95% CrI 0.47–0.79]), which is partly due to heterogeneity across the multiple surveys in Thailand. We calculated 90% prediction intervals for *O. viverrini* fecundity (figure 1*d*), which were validated using additional survey data where faecal egg counts and worm burdens were aggregated (TH5, LAO2 and LAO3; see table 1) and which were not used in our model fitting. We adjusted the reported mean worms expelled in surveys LAO2 and LAO3 by dividing by the recovery parameter *r* = 0.44, giving mean worm burdens of 25 and 6, respectively. The validation survey data are shown in figure 1*d*, and the relationships between worm burdens and faecal egg counts are within our 90% prediction intervals.

### (c) Parasite distribution and aggregation

Adult worm burdens of *O. viverrini* have been described as overdispersed within endemic populations. This motivated our fitting of the negative binomial distribution (equation (2.1)), with values of the aggregation parameter *k* below one indicating a significant departure from randomly distributed worms (Poisson variation). Our posterior mean estimates of the dispersion parameter *k*_*j*_ range from 0.24 to 0.56 by survey (figure 2*a*). Values of *k*_*j*_ across surveys were assumed to be normally distributed with a global mean (*μ*_*k*_) of 0.36 (95% CrI 0.12–0.55). Given our estimate of the standard deviation between surveys (*σ*_*k*_ = 0.18), our 90% prediction interval for *k* is 0.07–0.66. These values are indicative of high levels of parasite aggregation and are consistent with findings that most helminth species have *k* values between 0.1 and 1 [2,5,8]. Estimates for the mean *O. viverrini* burden by survey (*M*_*j*_) were all estimated to be higher than the observed mean flukes expelled (shown in table 1) and increased to 176 (TH1), 115 (TH2), 81 (TH3), 91 (TH4) and 245 (LAO1), respectively. The mean *O. viverrini* burden for the autopsy study (TH1) was estimated to be slightly higher than the recovered mean count of worms due to fitting the negative binomial distribution. Examining the relationship between values of *k* and mean worm burden by survey, we observe that parasite dispersion increases at lower worm burdens (figure 2*b*), as has also been reported for other helminths [54,55], presenting challenges for helminth elimination as parasites are aggregated in fewer hosts as control programmes progress.

### (d) Diagnostic sensitivity

We quantify the probability of observing parasite eggs for an individual as a function of the worm burden using a strictly increasing function; *se*(*x*) = *x*/(*b* + *x*). Given our mean estimate for the parameter *b* = 1.7 (95% CrI 1.6–1.9), the probability of observing at least one parasite egg when an individual is infected with one *O. viverrini* fluke is 0.37 (95% CrI 0.34–0.39), five flukes is 0.74 (95% CrI 0.72–0.76), 20 flukes is 0.92 (95% CrI 0.92–0.93), and reaches 0.99 at burdens of 115 flukes and higher, see figure 2*c*.

To calculate the sensitivity of faecal egg diagnostics at the population level, the probability of observing worms per individual is considered along with the distribution of adult worms (equation 2.4). Diagnostic sensitivity covaries with the adult worm burden, however, the relationship is nonlinear and depends on adult worm aggregation described by the negative binomial parameter *k* (figure 2*d*). Given the average worm dispersion observed in this study (*k* = 0.36), diagnostic sensitivity at a high mean worm burden, *M* = 100, is 0.88, reducing *M* to 20, 5, 1 or 0.1 results in population diagnostic sensitivities of 0.78, 0.66, 0.50 and 0.37, respectively. Therefore, when close to the threshold for elimination the proportion of false-negative cases is greater than or equal to 50%, which presents challenges both for case detection and the cost per detected case in low-intensity settings [56].

### (e) Prevalence

The relationship between the true prevalence of infection and the observed prevalence from faecal eggs is nonlinear and depends on the population diagnostic sensitivity; worm burden and aggregation; and assumptions on diagnostic specificity (equation 2.6). At an observed prevalence of 20%, which is the threshold for annual or biannual community deworming of *O. viverrini* [57], the true prevalence can vary from 29 to 42% depending on worm aggregation in the range *k* = 0.1–0.7 (figure 2*e*). Relaxing the assumption of perfect specificity for parasitological diagnosis has implications for interpreting observed parasite prevalence, with false-positive results dominating at low worm burdens (figure 2*f*).

## 4. Discussion

Control of helminths in humans is increasingly influenced by results from quantitative transmission models, which inform recommendations for national control programmes [29,58,59]. Predictions made by these models, such as the frequency and coverage of mass treatment required to achieve elimination, are strongly influenced by model structure and the assumed parameter values. This study brings together multiple datasets of individual-level parasite worm burdens and egg counts in conjunction with an epidemiological framework which quantifies key aspects of parasite ecology for the liver fluke *Opisthorchis viverrini*. Our estimates of the aggregation of adult worms, worm fecundity and diagnostic sensitivity as a function of worm burden (table 2) are essential for interpreting routinely collected parasitological survey data, understanding parasite transmission dynamics, and estimating the worm burden from indirect diagnostics [17,19]. Our inference framework is broadly applicably across helminth species and can be used to estimate epidemiological parameters for other parasites of medical and veterinary significance.

**Table 2.** Summary of epidemiological parameters for *Opisthorchis viverrini* estimated in this study. TH relates to parameters estimated from surveys in Thailand and LAO relates to surveys in Laos (table 1).

parameter	symbol	posterior mean	95% credible interval
worm aggregation	*μ*_*k*_	0.36	0.12–0.55
proportion of worms recovered by expulsion	*r*	0.44	0.42–0.45
egg output from 1 worm	*λ* (TH)	72	66–78
	*λ* (LAO)	20	13–30
density-dependence	*γ* (TH)	0.98	0.96–0.99
	*γ* (LAO)	0.82	0.74–0.90
egg dispersion	*h* (TH)	0.40	0.32–0.48
	*h* (LAO)	0.63	0.47–0.79

Macroparasite population dynamics are sensitive to small differences in the aggregation of adult worms, given by the negative binomial parameter *k* [2,60]. Here, we have quantified *k* and its variation between surveys using a hierarchical model which pools information between five surveys, making the posterior estimates robust to outlier values. An analysis of *O. viverrini* transmission dynamics in Southern Laos assumed that the negative binomial dispersion of adult worms given by the parameter *k* was 0.1 [61]. Given our prediction interval for *k*, we estimate there is a 93% probability that the value of *k* is higher than 0.1, and our best estimate is 0.36. However, given the strong observed relationship between mean worm burden and *k* [54,55], a value of 0.1 is plausible in lower-intensity settings (figure 2*b*). Parasite aggregation is caused by heterogeneity in host exposure and/or susceptibility. For *O. viverrini* aggregated worm burdens are ultimately driven by variance in individual feeding rates and the density of metacercariae in local fish [30]. There is a cultural component to transmission as consumption of raw fish is perceived to be linked with male virility and is a marker for local identity and traditional practices in the Northeast ‘Isan’ region of Thailand [62,63]. The distribution of *O. viverrini* metacercariae in cyprinid fish intermediate hosts is also overdispersed [33], which further contributes to variance in the adult worm distribution within definitive human hosts; however, this has not yet been quantified by fitting the negative binomial distribution.

The relationship between mature worms and indirect diagnostics, such as faecal egg counts, is central to estimating the worm burden and describing transmission potential. Our models support different patterns of helminth fecundity in surveys from Thailand over the period 1966–1992 versus more recent surveys from Laos conducted between 2005 and 2011 (figure 1). These findings bolster the claim that helminth fecundity varies between host populations, as previously reported for *A. lumbricoides* [14]. The mechanisms for these differences are poorly understood and may comprise multiple host and parasite factors, however ecological studies of helminths show an important role for host nutrition [64].

Imperfect diagnostic observations bias estimates of epidemiological parameters [44]. Here, we explicitly quantify these inaccuracies by estimating the proportion of worms recovered by worm expulsion and the probability of detecting faecal eggs as a function of individual worm burden. Our sensitivity estimates at the population level (figure 2*d*) are most relevant for the formalin–ether concentration technique (FECT) used in the majority of surveys (table 1) and we accounted for the alternative diagnostic used in survey TH2 (Methods). Our results at lower mean worm burdens are consistent with estimates of FECT sensitivity validated with PCR and worm expulsion as the gold standard [65]. Understanding how diagnostic sensitivity changes in response to a declining worm burden has important implications for control programmes as the number of false negatives increases when a programme is close to elimination and so larger sample sizes are required to estimate endemicity. There is a nonlinear relationship between the observed and true prevalence, which is dependent on the population worm burden and the degree of aggregation (figure 2*e* and equation (2.6)). At an observed prevalence of 20% for *O. viverrini*, below which the World Health Organization advises mass drug administration to reduce from biannual to annual treatment [57], we estimate that the true prevalence is actually twofold higher at 40% where *k* > 0.5. These findings demonstrate that prevalence of infection is an unreliable indicator on which to base thresholds for helminth control programmes.

There were limitations to our analysis. Recruitment in expulsion surveys was not always random and if individuals with low or zero egg counts are underrepresented in the sample, this may influence our estimation of sensitivity, as there are fewer observations from individuals with low worm burdens, and inflate values of *k*, as the community worm burden appears more homogeneous; although by using partial pooling we likely reduced the bias in estimating *k*. An additional challenge is the presence of minute intestinal flukes; helminths with the same transmission route as *O. viverrini* which frequently occur as co-infections [39,65]. These flukes produce visually similar eggs to *O. viverrini* leading to imperfect specificity by parasitological diagnostics. Our ability to account for misdiagnosis in model fitting was limited as most surveys in Thailand did not clarify whether intestinal flukes were present. We explore the impact of imperfect diagnostic specificity on the observed prevalence under simplistic scenarios shown in figure 2*f*; in reality, we expect specificity for *O. viverrini* to covary with the endemicity of minute intestinal flukes. The increased use of molecular diagnostics enables the species of parasite egg to be identified [66], and recent improvements to *O. viverrini* antigen tests also provides a method to discriminate cases [67,68].

Future studies on macroparasite epidemiology would benefit from a renewed focus on the worm burden to glean insights into transmission dynamics, parasite-induced morbidity, the impact of interventions, and the efficacy of anthelmintic treatment. For species where it is difficult to observe worm burdens directly, population genetics also provides a means to assess the relatedness between offspring and thus estimate the number of mature adult worms [69,70].

Research on the epidemiology of parasitic helminths experienced a period of substantial development between roughly 1978–2000, instigated by the collaboration between Roy Anderson and Robert May who drew together ideas from ecology, parasitology and mathematics into a highly influential series of deterministic models [1–3]. This framework for infectious diseases was further developed by a generation of researchers, many of whom were graduate students or postdocs of Anderson & May [71]. In recent years, the epidemiology of microparasites has advanced significantly, driven by both theoretical work [4] and improved data (in particular, whole-genome sequencing makes it possible to infer transmission networks for certain pathogens [72,73]); however, research on macroparasites has received less attention [74,75]. Many scientific questions raised in foundational studies, including the extent to which adult worm overdispersion is driven by host or parasite genetic factors, have arguably remained unanswered and the focus on worm burdens has been neglected by many in the field with insights from disease ecology replaced by less appropriate techniques from medical statistics [43,76]. The literature is replete with criticisms of ‘classical’ frequentist statistics and null-hypothesis testing [77–79]. While reiterating these arguments goes beyond the scope of this article, we emphasize that estimating biologically meaningful parameters is a powerful framework for understanding parasite ecology.

In this study, we have quantified the distribution of adult worms in host populations and worm fecundity, and in doing so provide a inference framework to convert readily available diagnostic data into worm burdens to further our understanding of the transmission, evolution and control of helminth parasites.

## Data Availability

Data used for model fitting (individual worm and egg counts) are available from Dryad https://doi.org/10.5061/dryad.q83bk3jn6 [80]. Code to reproduce the analysis is available at the GitHub repository https://github.com/tc13/worm-inference.
